# 2-[2-(1*H*-indol-3-yl)ethyl­iminiomethyl]-4-nitro­phenolate

**DOI:** 10.1107/S1600536808011185

**Published:** 2008-04-26

**Authors:** Hapipah M. Ali, M. I. Mohamed Mustafa, M. Razali Rizal, Seik Weng Ng

**Affiliations:** a Department of Chemistry, University of Malaya, 50603 Kuala Lumpur, Malaysia

## Abstract

The title Schiff base, C_17_H_15_N_3_O_3_, exists in the zwitterionic form with the phenol H atom transferred to the imine group. Adjacent zwitterions are linked into a linear chain running along the *a* axis by an indole–hydr­oxy N—H⋯O hydrogen bond [3.100 (2) Å].

## Related literature

For the structure of the zwitterionic 2-{[3-(indol-3-yl)propen­yl]methyl­ammonio}-4-methyl­phenolate, see: Ali *et al.* (2007 ▶).
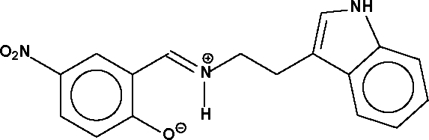


## Experimental

### 

#### Crystal data

C_17_H_15_N_3_O_3_
*M* *_r_* = 309.32
Monoclinic, 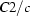

*a* = 14.5990 (7) Å
*b* = 9.5027 (5) Å
*c* = 21.5373 (10) Å
β = 95.712 (2)°
*V* = 2973.0 (3) Å^3^
*Z* = 8
Mo *K*α radiation
μ = 0.10 mm^−1^
*T* = 139 (2) K
0.51 × 0.30 × 0.19 mm

#### Data collection

Bruker SMART APEX diffractometer
Absorption correction: none
6383 measured reflections
3312 independent reflections
2403 reflections with *I* > 2σ(*I*)
*R*_int_ = 0.023

#### Refinement

*R*[*F* ^2^ > 2σ(*F* ^2^)] = 0.049
*wR*(*F* ^2^) = 0.161
*S* = 1.06
3312 reflections
216 parameters
2 restraints
H atoms treated by a mixture of independent and constrained refinement
Δρ_max_ = 1.18 e Å^−3^
Δρ_min_ = −0.27 e Å^−3^

### 

Data collection: *APEX2* (Bruker, 2005 ▶); cell refinement: *SAINT* (Bruker, 2005 ▶); data reduction: *SAINT*; program(s) used to solve structure: *SHELXS97* (Sheldrick, 2008 ▶); program(s) used to refine structure: *SHELXL97* (Sheldrick, 2008 ▶); molecular graphics: *X-SEED* (Barbour, 2001 ▶); software used to prepare material for publication: *publCIF* (Westrip, 2008 ▶).

## Supplementary Material

Crystal structure: contains datablocks global, I. DOI: 10.1107/S1600536808011185/bv2090sup1.cif

Structure factors: contains datablocks I. DOI: 10.1107/S1600536808011185/bv2090Isup2.hkl

Additional supplementary materials:  crystallographic information; 3D view; checkCIF report

## Figures and Tables

**Table 1:** Hydrogen-bond geometry (Å, °)

*D*—H⋯*A*	*D*—H	H⋯*A*	*D*⋯*A*	*D*—H⋯*A*
N2—H2n⋯O1	0.88 (1)	1.87 (2)	2.602 (2)	139 (2)
N3—H3n⋯O2^i^	0.88 (1)	2.36 (2)	3.027 (2)	133 (2)
N3—H3n⋯O3^i^	0.88 (1)	2.23 (1)	3.100 (2)	171 (2)

Symmetry code: (i) 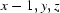.

**Fig. 1.**
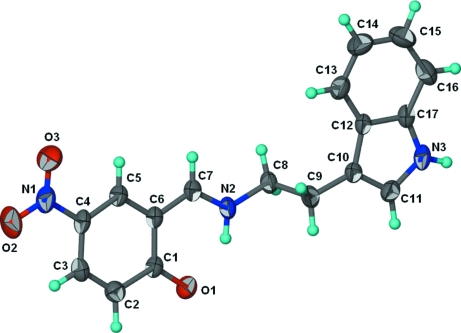
Thermal ellipsoid plot of C17H15N3O3. Displacement ellipsoids are drawn at the 70% probability level, and H atoms are shown as spheres of arbitrary radii.

## supplementary crystallographic information

### Experimental

Tryptamine (0.32 g, 2 mmol) and 5-nitrosalisylaldehyde (0.33 g, 21.9 mmol) were refluxed in ethanol (50 ml) for 2 h. The solvent was removed to give the product Schiff base, and crystals were obtained by recrystallization from THF.

### Refinement

The carbon-bound H atoms were placed at calculated positions (C–H 0.95 Å), and were included in the refinement in the riding model approximation with *U*(H) set to 1.2*U*_eq_(C). The amino hydrogen atom was located in a difference Fouier map, and was refined with a distance restraint of N–H 0.88±0.01 Å.

The final difference Fourier map had a large peak at 1.5 Å from O1 and H2*n*. This peak is not near the the nitro group even though this group has larger thermal parameters than the rest of the molecule.

### Crystal data

**Table:**

C_17_H_15_N_3_O_3_	*F*(000) = 1296
*M**_r_* = 309.32	*D*_x_ = 1.382 Mg m^−^^3^
Monoclinic, *C*2/*c*	Mo *K*α radiation, λ = 0.71073 Å
Hall symbol: -c 2yc	Cell parameters from 2068 reflections
*a* = 14.5990 (7) Å	θ = 5.1–59.5°
*b* = 9.5027 (5) Å	µ = 0.10 mm^−^^1^
*c* = 21.5373 (10) Å	*T* = 139 K
β = 95.712 (2)°	Irregular, yellow
*V* = 2973.0 (3) Å^3^	0.51 × 0.30 × 0.19 mm
*Z* = 8	

### Data collection

**Table:**

Bruker APEXII diffractometer	2403 reflections with *I* > 2σ(*I*)
Radiation source: medium-focus sealed tube	*R*_int_ = 0.023
graphite	θ_max_ = 27.5°, θ_min_ = 1.9°
φ and ω scans	*h* = −14→18
6383 measured reflections	*k* = −12→9
3312 independent reflections	*l* = −27→26

### Refinement

**Table:**

Refinement on *F*^2^	Primary atom site location: structure-invariant direct methods
Least-squares matrix: full	Secondary atom site location: difference Fourier map
*R*[*F*^2^ > 2σ(*F*^2^)] = 0.049	Hydrogen site location: inferred from neighbouring sites
*wR*(*F*^2^) = 0.161	H atoms treated by a mixture of independent and constrained refinement
*S* = 1.06	*w* = 1/[σ^2^(*F*_o_^2^) + (0.0911*P*)^2^ + 1.065*P*] where *P* = (*F*_o_^2^ + 2*F*_c_^2^)/3
3312 reflections	(Δ/σ)_max_ = 0.001
216 parameters	Δρ_max_ = 1.18 e Å^−^^3^
2 restraints	Δρ_min_ = −0.27 e Å^−^^3^

### Special details

**Table:**

Geometry. All e.s.d.'s (except the e.s.d. in the dihedral angle between two l.s. planes) are estimated using the full covariance matrix. The cell e.s.d.'s are taken into account individually in the estimation of e.s.d.'s in distances, angles and torsion angles; correlations between e.s.d.'s in cell parameters are only used when they are defined by crystal symmetry. An approximate (isotropic) treatment of cell e.s.d.'s is used for estimating e.s.d.'s involving l.s. planes.

### Fractional atomic coordinates and isotropic or equivalent isotropic displacement parameters (Å^2^)

**Table:**

	*x*	*y*	*z*	*U*_iso_*/*U*_eq_	
O1	0.60196 (9)	0.52975 (15)	0.49373 (6)	0.0348 (3)	
O2	1.01389 (10)	0.6546 (2)	0.56727 (9)	0.0596 (5)	
O3	0.95763 (10)	0.75509 (18)	0.64477 (7)	0.0501 (5)	
N1	0.94781 (11)	0.68697 (19)	0.59563 (8)	0.0377 (4)	
N2	0.53650 (11)	0.61992 (16)	0.59452 (7)	0.0278 (4)	
N3	0.16678 (11)	0.81046 (17)	0.64386 (8)	0.0308 (4)	
C1	0.68118 (12)	0.56415 (18)	0.51803 (8)	0.0254 (4)	
C2	0.76150 (13)	0.54714 (19)	0.48512 (8)	0.0286 (4)	
H2	0.7547	0.5085	0.4442	0.034*	
C3	0.84661 (13)	0.58457 (19)	0.51059 (9)	0.0283 (4)	
H3	0.8986	0.5705	0.4880	0.034*	
C4	0.85759 (12)	0.64418 (19)	0.57044 (9)	0.0273 (4)	
C5	0.78428 (12)	0.66384 (18)	0.60489 (8)	0.0258 (4)	
H5	0.7935	0.7037	0.6455	0.031*	
C6	0.69633 (12)	0.62481 (18)	0.57975 (8)	0.0241 (4)	
C7	0.62047 (12)	0.65009 (18)	0.61477 (8)	0.0258 (4)	
H7	0.6321	0.6911	0.6550	0.031*	
C8	0.45617 (12)	0.6528 (2)	0.62664 (9)	0.0285 (4)	
H8A	0.4761	0.6835	0.6698	0.034*	
H8B	0.4177	0.5675	0.6288	0.034*	
C9	0.39951 (13)	0.7694 (2)	0.59244 (9)	0.0303 (4)	
H9A	0.4375	0.8556	0.5918	0.036*	
H9B	0.3823	0.7402	0.5487	0.036*	
C10	0.31404 (12)	0.80148 (19)	0.62293 (8)	0.0253 (4)	
C11	0.22563 (13)	0.77101 (19)	0.60122 (9)	0.0298 (4)	
H11	0.2072	0.7284	0.5621	0.036*	
C12	0.21685 (12)	0.86790 (19)	0.69435 (9)	0.0269 (4)	
C13	0.18875 (15)	0.9262 (2)	0.74883 (10)	0.0391 (5)	
H13	0.1259	0.9262	0.7567	0.047*	
C14	0.25584 (18)	0.9837 (3)	0.79057 (10)	0.0482 (6)	
H14	0.2387	1.0238	0.8281	0.058*	
C15	0.34794 (18)	0.9848 (2)	0.77940 (10)	0.0467 (6)	
H15	0.3923	1.0267	0.8090	0.056*	
C16	0.37574 (14)	0.9260 (2)	0.72603 (9)	0.0349 (5)	
H16	0.4389	0.9265	0.7188	0.042*	
C17	0.30999 (12)	0.86553 (18)	0.68270 (8)	0.0240 (4)	
H2N	0.5301 (16)	0.578 (2)	0.5578 (6)	0.047 (7)*	
H3N	0.1078 (8)	0.790 (3)	0.6400 (11)	0.052 (7)*	

### Atomic displacement parameters (Å^2^)

**Table:**

	*U*^11^	*U*^22^	*U*^33^	*U*^12^	*U*^13^	*U*^23^
O1	0.0221 (7)	0.0421 (8)	0.0390 (8)	−0.0044 (6)	−0.0020 (6)	−0.0080 (6)
O2	0.0177 (8)	0.0831 (13)	0.0789 (12)	−0.0053 (8)	0.0097 (8)	−0.0235 (10)
O3	0.0286 (9)	0.0690 (12)	0.0514 (9)	−0.0109 (8)	−0.0022 (7)	−0.0173 (8)
N1	0.0197 (9)	0.0436 (10)	0.0493 (10)	−0.0028 (7)	0.0012 (7)	−0.0020 (8)
N2	0.0194 (8)	0.0308 (8)	0.0334 (8)	0.0039 (6)	0.0040 (6)	−0.0015 (6)
N3	0.0162 (8)	0.0303 (8)	0.0452 (9)	−0.0007 (6)	0.0001 (7)	−0.0016 (7)
C1	0.0211 (9)	0.0229 (8)	0.0316 (9)	0.0007 (7)	−0.0006 (7)	0.0002 (7)
C2	0.0290 (10)	0.0274 (9)	0.0298 (9)	0.0004 (8)	0.0041 (8)	−0.0010 (7)
C3	0.0229 (9)	0.0273 (9)	0.0357 (10)	0.0014 (7)	0.0076 (7)	0.0024 (8)
C4	0.0164 (9)	0.0269 (9)	0.0378 (10)	−0.0005 (7)	−0.0007 (7)	0.0025 (7)
C5	0.0216 (9)	0.0243 (9)	0.0311 (9)	−0.0001 (7)	0.0004 (7)	0.0001 (7)
C6	0.0194 (9)	0.0224 (8)	0.0305 (9)	0.0013 (7)	0.0020 (7)	0.0017 (7)
C7	0.0225 (10)	0.0241 (8)	0.0305 (9)	0.0029 (7)	0.0013 (7)	0.0016 (7)
C8	0.0202 (10)	0.0343 (10)	0.0319 (9)	0.0042 (7)	0.0069 (7)	0.0022 (7)
C9	0.0269 (10)	0.0329 (10)	0.0319 (9)	0.0082 (8)	0.0063 (8)	0.0027 (8)
C10	0.0226 (9)	0.0255 (8)	0.0275 (9)	0.0044 (7)	0.0012 (7)	0.0012 (7)
C11	0.0281 (10)	0.0276 (9)	0.0323 (9)	0.0039 (8)	−0.0047 (8)	−0.0027 (7)
C12	0.0209 (9)	0.0258 (9)	0.0341 (9)	0.0029 (7)	0.0031 (7)	0.0033 (7)
C13	0.0362 (12)	0.0413 (11)	0.0422 (11)	0.0092 (9)	0.0162 (9)	0.0023 (9)
C14	0.0619 (16)	0.0498 (13)	0.0336 (11)	0.0158 (12)	0.0087 (11)	−0.0085 (10)
C15	0.0499 (14)	0.0478 (13)	0.0388 (11)	0.0072 (11)	−0.0129 (10)	−0.0144 (10)
C16	0.0262 (10)	0.0361 (10)	0.0406 (11)	0.0029 (8)	−0.0063 (8)	−0.0047 (8)
C17	0.0190 (9)	0.0239 (8)	0.0286 (9)	0.0034 (7)	0.0005 (7)	0.0006 (7)

### Geometric parameters (Å, °)

**Table:**

O1—C1	1.264 (2)	C7—H7	0.9500
O2—N1	1.231 (2)	C8—C9	1.527 (3)
O3—N1	1.237 (2)	C8—H8A	0.9900
N1—C4	1.433 (2)	C8—H8B	0.9900
N2—C7	1.292 (2)	C9—C10	1.498 (2)
N2—C8	1.454 (2)	C9—H9A	0.9900
N2—H2N	0.883 (10)	C9—H9B	0.9900
N3—C12	1.363 (3)	C10—C11	1.359 (3)
N3—C11	1.371 (2)	C10—C17	1.430 (2)
N3—H3N	0.879 (10)	C11—H11	0.9500
C1—C2	1.439 (2)	C12—C13	1.396 (3)
C1—C6	1.446 (3)	C12—C17	1.407 (2)
C2—C3	1.355 (3)	C13—C14	1.375 (3)
C2—H2	0.9500	C13—H13	0.9500
C3—C4	1.402 (3)	C14—C15	1.389 (4)
C3—H3	0.9500	C14—H14	0.9500
C4—C5	1.375 (2)	C15—C16	1.375 (3)
C5—C6	1.393 (3)	C15—H15	0.9500
C5—H5	0.9500	C16—C17	1.394 (3)
C6—C7	1.421 (2)	C16—H16	0.9500
			
O2—N1—O3	121.71 (18)	N2—C8—H8B	109.5
O2—N1—C4	118.51 (17)	C9—C8—H8B	109.5
O3—N1—C4	119.78 (16)	H8A—C8—H8B	108.1
C7—N2—C8	125.09 (16)	C10—C9—C8	111.78 (14)
C7—N2—H2N	114.5 (16)	C10—C9—H9A	109.3
C8—N2—H2N	120.4 (16)	C8—C9—H9A	109.3
C12—N3—C11	108.79 (15)	C10—C9—H9B	109.3
C12—N3—H3N	127.4 (16)	C8—C9—H9B	109.3
C11—N3—H3N	123.3 (16)	H9A—C9—H9B	107.9
O1—C1—C2	121.60 (16)	C11—C10—C17	106.10 (15)
O1—C1—C6	122.27 (16)	C11—C10—C9	127.56 (17)
C2—C1—C6	116.12 (16)	C17—C10—C9	126.29 (17)
C3—C2—C1	122.05 (17)	C10—C11—N3	110.38 (16)
C3—C2—H2	119.0	C10—C11—H11	124.8
C1—C2—H2	119.0	N3—C11—H11	124.8
C2—C3—C4	119.64 (16)	N3—C12—C13	130.60 (18)
C2—C3—H3	120.2	N3—C12—C17	107.60 (15)
C4—C3—H3	120.2	C13—C12—C17	121.76 (19)
C5—C4—C3	121.81 (17)	C14—C13—C12	117.22 (19)
C5—C4—N1	119.53 (17)	C14—C13—H13	121.4
C3—C4—N1	118.65 (16)	C12—C13—H13	121.4
C4—C5—C6	119.41 (17)	C13—C14—C15	121.91 (19)
C4—C5—H5	120.3	C13—C14—H14	119.0
C6—C5—H5	120.3	C15—C14—H14	119.0
C5—C6—C7	119.05 (16)	C16—C15—C14	120.9 (2)
C5—C6—C1	120.95 (16)	C16—C15—H15	119.6
C7—C6—C1	119.97 (16)	C14—C15—H15	119.6
N2—C7—C6	123.14 (17)	C15—C16—C17	119.08 (19)
N2—C7—H7	118.4	C15—C16—H16	120.5
C6—C7—H7	118.4	C17—C16—H16	120.5
N2—C8—C9	110.52 (14)	C16—C17—C12	119.15 (17)
N2—C8—H8A	109.5	C16—C17—C10	133.65 (16)
C9—C8—H8A	109.5	C12—C17—C10	107.12 (16)
			
O1—C1—C2—C3	179.54 (17)	C8—C9—C10—C11	−108.7 (2)
C6—C1—C2—C3	0.6 (3)	C8—C9—C10—C17	68.3 (2)
C1—C2—C3—C4	−1.2 (3)	C17—C10—C11—N3	−0.7 (2)
C2—C3—C4—C5	1.2 (3)	C9—C10—C11—N3	176.80 (17)
C2—C3—C4—N1	−178.08 (17)	C12—N3—C11—C10	0.1 (2)
O2—N1—C4—C5	172.08 (18)	C11—N3—C12—C13	178.3 (2)
O3—N1—C4—C5	−8.1 (3)	C11—N3—C12—C17	0.5 (2)
O2—N1—C4—C3	−8.7 (3)	N3—C12—C13—C14	−176.5 (2)
O3—N1—C4—C3	171.20 (18)	C17—C12—C13—C14	1.0 (3)
C3—C4—C5—C6	−0.5 (3)	C12—C13—C14—C15	0.2 (3)
N1—C4—C5—C6	178.68 (16)	C13—C14—C15—C16	−0.9 (4)
C4—C5—C6—C7	−177.79 (16)	C14—C15—C16—C17	0.4 (3)
C4—C5—C6—C1	−0.1 (3)	C15—C16—C17—C12	0.8 (3)
O1—C1—C6—C5	−178.92 (17)	C15—C16—C17—C10	177.4 (2)
C2—C1—C6—C5	0.1 (2)	N3—C12—C17—C16	176.45 (17)
O1—C1—C6—C7	−1.2 (3)	C13—C12—C17—C16	−1.5 (3)
C2—C1—C6—C7	177.77 (16)	N3—C12—C17—C10	−0.9 (2)
C8—N2—C7—C6	−175.39 (16)	C13—C12—C17—C10	−178.93 (18)
C5—C6—C7—N2	178.55 (16)	C11—C10—C17—C16	−175.9 (2)
C1—C6—C7—N2	0.8 (3)	C9—C10—C17—C16	6.6 (3)
C7—N2—C8—C9	109.1 (2)	C11—C10—C17—C12	1.0 (2)
N2—C8—C9—C10	177.58 (15)	C9—C10—C17—C12	−176.53 (17)

### Hydrogen-bond geometry (Å, °)

**Table:**

*D*—H···*A*	*D*—H	H···*A*	*D*···*A*	*D*—H···*A*
N2—H2n···O1	0.88 (1)	1.87 (2)	2.602 (2)	139 (2)
N3—H3n···O2^i^	0.88 (1)	2.36 (2)	3.027 (2)	133 (2)
N3—H3n···O3^i^	0.88 (1)	2.23 (1)	3.100 (2)	171 (2)

Symmetry codes: (i) *x*−1, *y*, *z*.
